# Evidence That Adrenergic Ventrolateral Medullary Cells Are Activated whereas Precerebellar Lateral Reticular Nucleus Neurons Are Suppressed during REM Sleep

**DOI:** 10.1371/journal.pone.0062410

**Published:** 2013-04-22

**Authors:** Georg M. Stettner, Yanlin Lei, Kate Benincasa Herr, Leszek Kubin

**Affiliations:** Department of Animal Biology, School of Veterinary Medicine, University of Pennsylvania, Philadelphia, Pennsylvania, United States of America; Imperial College London, United Kingdom

## Abstract

Rapid eye movement sleep (REMS) is generated in the brainstem by a distributed network of neurochemically distinct neurons. In the pons, the main subtypes are cholinergic and glutamatergic REMS-on cells and aminergic REMS-off cells. Pontine REMS-on cells send axons to the ventrolateral medulla (VLM), but little is known about REMS-related activity of VLM cells. In urethane-anesthetized rats, dorsomedial pontine injections of carbachol trigger REMS-like episodes that include cortical and hippocampal activation and suppression of motoneuronal activity; the episodes last 4–8 min and can be elicited repeatedly. We used this model to determine whether VLM catecholaminergic cells are silenced during REMS, as is typical of most aminergic neurons studied to date, and to investigate other REMS-related cells in this region. In 18 anesthetized, paralyzed and artificially ventilated rats, we obtained extracellular recordings from VLM cells when REMS-like episodes were elicited by pontine carbachol injections (10 mM, 10 nl). One major group were the cells that were activated during the episodes (n = 10). Their baseline firing rate of 3.7±2.1 (SD) Hz increased to 9.7±2.1 Hz. Most were found in the adrenergic C1 region and at sites located less than 50 µm from dopamine β-hydroxylase-positive (DBH^+^) neurons. Another major group were the silenced or suppressed cells (n = 35). Most were localized in the lateral reticular nucleus (LRN) and distantly from any DBH^+^ cells. Their baseline firing rates were 6.8±4.4 Hz and 15.8±7.1 Hz, respectively, with the activity of the latter reduced to 7.4±3.8 Hz. We conclude that, in contrast to the pontine noradrenergic cells that are silenced during REMS, medullary adrenergic C1 neurons, many of which drive the sympathetic output, are activated. Our data also show that afferent input transmitted to the cerebellum through the LRN is attenuated during REMS. This may distort the spatial representation of body position during REMS.

## Introduction

Rapid eye movement sleep (REMS) is a state characterized by wake-like activation of the cortex and hippocampus accompanied by a loss of activity in postural muscles (atonia) and a host of phasic phenomena, such as rapid eye movements, twitches of the distal limb and orofacial muscles, and variable breathing and arterial blood pressure [1]. REMS is the state when dreams occur and it plays an important role in brain development and processing of memories acquired during the waking states [2], [3], [4]. It is also a state whose expression characteristically changes with aging and neurodegenerative disorders [5], [6], [7], [8]. For all of these reasons, extensive efforts have been invested in studies of the neural mechanisms and networks responsible for the generation and modulation of this phase of sleep.

Although expression of REMS is dependent on modulatory influences exerted by the forebrain, the brainstem is the principal site of origin of the state [9], [10]. Single cell recordings obtained from the pons indicated the presence of two major cell types likely to play a key role in the generation of REMS: the REMS-on cells that are tonically activated in association with the occurrence of REMS episodes and REMS-off cells that are suppressed or silenced in a reciprocal manner relative to the activity of the REMS-on neurons. The pontine REMS-on cells include cholinergic and glutamatergic neurons, whereas the best identified pontine REMS-off cells are those containing serotonin (dorsal raphe nucleus) and norepinephrine (locus coeruleus (LC)), also designated as the A6 noradrenergic group) [11], [12], [13], [14], [15], [16], [17], [18], [19]. Based on these findings, a reciprocal cholinergic-aminergic network model has been proposed to explain the generation of REMS [20], and it was subsequently modified to include pontine excitatory glutamatergic and inhibitory (GABA-ergic) neurons [21]. However, further tests and refinements of the existing models are needed to advance our understanding of the mechanisms responsible for the generation of REMS and, ultimately, to understand its physiologic role.

Pontine REMS-related cells interact with many locally and remotely located targets and this interaction determines the timing of REMS occurrence within the sleep-wake cycle, and presumably also the impact of REMS on brain functions (reviewed in [20], [21], [22], [23], [24]). In particular, the connections between REMS-related cells in the pons and those located in the medullary reticular formation [25], [26], [27], [28] appear to be very important because REMS is severely curtailed or abolished following certain medullary lesions or when the connections between the pons and medulla are interrupted [29], [30]. Thus, the interactions between the pontine and medullary reticular formation cells with REMS-related activity need to be elucidated to fully understand the key elements of the brainstem network responsible for the generation of REMS and its characteristic phenomena.

To date, studies of REMS-related cells in the medulla lag behind the corresponding studies in the pons. This is due, in part, to historically greater attention paid to the pontine mechanisms but the progress is also hampered by the technical difficulty to record cell activities across the sleep-wake cycle at sites located close to the highly mobile spino-medullary junction. Nevertheless, cell recordings in chronically instrumented cats demonstrated that the medial reticular formation of the rostral medulla contains appreciable numbers of REMS-on neurons [31], [32], [33], [34], [35] and that serotonergic cells located along the medullary midline have REMS-off firing patterns [36], [37], [38]. However, the studies in chronically instrumented, behaving animals are limited in that the locations of the recording sites often cannot be precisely determined and the neurochemical identity of the recorded neurons is difficult to ascertain. In addition, no recordings were conducted to date from more caudal regions of the ventrolateral medulla (VLM) due to accessibility problems in behaving animals. Consequently, inferences about the specific role of medullary REMS-related cells have been often based on juxtaposition of results collected from behaving animals with anatomical data separately obtained by means of tract tracing and immunohistochemistry. Thus, with the exception of caudal medullary serotonergic neurons [36], [37], [38], [39] and rostromedial medullary reticular neurons [34], comprehensive evidence associating REMS-related cellular activity in the medulla to the neurochemical identity of the recorded cells and/or their functions is not available. Nevertheless, compelling suggestions have been made based on indirect evidence. For example, it has been proposed that VLM REMS-on neurons are inhibitory, possibly GABA-ergic, whereas the catecholaminergic A1 and C1 neurons are of the REMS-off type [40], [41].

Within the caudal and intermediate VLM, there are three major groups of cells whose functions and connectivity have been well characterized but whose behavior during REMS is unknown. At relatively rostral levels, there are the adrenergic cells of the C1 group, many of which drive the sympathetic output and have extensive connections within the brainstem [42], [43], [44], [45], [46], [47], [48], [49], [50]. Further caudal, the VLM contains noradrenergic cells (A1 group) that may contribute to cardiovascular and pain regulations [51], [52], [53], [54], [55] through their axonal projections to the brainstem and spinal cord [56], [57]. They mainly aggregate just dorsal and dorsomedial to the lateral reticular nucleus (LRN), a major precerebellar structure that integrates descending motor commands with vestibular information and flexor reflex afferents ascending from the spinal cord [58], [59], [60], [61], [62], [63], [64].

REMS is associated with a major reconfiguration of both cardiorespiratory control and central processing of sensorymotor information and yet, the effects of REMS on activity of C1, A1 and LRN neurons have not been characterized. Thus, our goal was to determine the effect of REMS on the activity of cells in the VLM in relation to their neurochemical and/or functional phenotypes. To achieve this, we used a pharmacological model of REMS that allows one to effectively sample single cell activity and unimpeded access to the caudal VLM. We used urethane-anesthetized rats in which dorsomedial pontine injections of carbachol trigger REMS-like episodes that include cortical and hippocampal activation and suppression of activity in hypoglossal motoneurons [65], [66]. The REMS-like episodes elicited in this model last 4–8 min and can be triggered repeatedly, thus making the model particularly suitable for observation of electrophysiological activity of single cells in a well-controlled experimental setting. We have previously validated this model as adequately representing multiple tonic features of natural REMS. This included a demonstration that the effective regions for microinjections of both carbachol and the GABA_A_ receptor antagonist, bicuculline, into the dorsal pontine tegmentum are well defined [67] and correspond to the effective regions in behaving rats [68], [69], [70], [71]. We determined that the pontine noradrenergic LC neurons and caudal medullary serotonergic neurons are silenced during the REMS-like episodes elicited by pontine microinjections of carbachol [39], [65], as they are during natural REMS [13], [37]. Furthermore, we determined that activation of neurons in the wake-related posterior, lateral hypothalamus suppresses the ability of pontine carbachol to trigger REMS-like episodes [66]. Similarly, in behaving animals, activation of cells in this hypothalamic region suppresses sleep and awakens the animal [72], [73]. Thus, while the model is limited in that it does not generate phasic events of REMS (see [74], [65], [75] for discussion), it otherwise mimics at many levels the processes underlying the initiation and maintenance of REMS.

Our present extracellular recordings from caudal VLM neurons combined with immunohistochemistry and complemented with analysis of cell action potentials characteristics and firing patterns reveal that adrenergic VLM cells (C1 group) are activated whereas the precerebellar LRN neurons are silenced or suppressed during REMS-like episodes. These findings extend our understanding of the mechanisms underlying the medullary contribution to the generation of REMS and the impact of REMS on the cardiovascular and somatosensory systems. They also prompt a reinterpretation of prior cellular studies of REMS-related cells in the VLM. A preliminary report has been published [76].

## Materials and Methods

### Ethics Statement

All animal procedures followed the guidelines of the *Guide for the Care and Use of Laboratory Animals* of the National Institutes of Health and were approved by the Institutional Animal Care and Use Committee of the University of Pennsylvania (Protocol no. 803882). All experimental procedures were performed under anesthesia and with continuous monitoring of electroencephalogram (EEG), respiratory activity and blood pressure to ensure stable and pain-free conditions.

### Animal Preparation, Microinjection and Recording Techniques

Experiments were performed on 18 adult, male Sprague-Dawley rats (390±19 (SD) g body weight) obtained from Charles River Laboratories (Wilmington, MA).

Rats were pre-anesthetized with isoflurane (3%) followed by urethane (1.0 g/kg i.v. via a tail vein catheter). They were tracheotomized and had a femoral artery and vein catheterized for arterial blood pressure monitoring and fluid/drug administration, respectively. The medial branch of the right XII nerve was dissected, cut and its central end was placed in a cuff-type recording electrode (modified after [77]). Both cervical vagi were cut to enhance XII nerve activity and make it independent of lung volume feedback. The animal’s head was placed in a stereotaxic holder and openings were made in the parietal bone on the left side for inserting a carbachol-containing pipette into the dorsomedial pons and on the right side for inserting a bipolar recording electrode into the hippocampus. The atlanto-occipital membrane was exposed, the caudal edge of the occipital bone was removed, and the dura and pia matters overlying the cerebellar vermis and medulla were cut and retracted laterally to allow for insertion of a recording pipette into the VLM. Two screws were attached to the skull (2 mm anterior and 2 mm left/right from bregma) to record cortical EEG. Hippocampal activity was recorded using an electrode constructed from two Teflon-insulated platinum wires (Model 771000; A-M Systems, Sequim, WA) with tips separated by 0.8 mm. The electrode was inserted 3.7 mm posterior to bregma, 2.2 mm right from the midline and 2.4 mm below the cortical surface.

The animals were paralyzed with pancuronium bromide (1 mg/kg i.v., Sigma, St. Louis, MO) and artificially ventilated with an air-oxygen mixture (30–60% O_2_). The central respiratory drive was set by first ventilating the animal to the apneic threshold and then gradually reducing the tidal volume of the ventilator until a steady respiratory modulation of XII nerve activity was established. Subsequently, the rate and volume of artificial ventilation were kept constant. Adequate level of anesthesia was verified based on stability of the amplitude and constant rate of inspiratory bursts recorded from the XII nerve, stable heart rate and arterial blood pressure, and stable cortical EEG and hippocampal activities. If needed, supplemental doses of urethane were administered in 0.2–0.3 mg/kg increments. Adequate paralysis was maintained by continuous infusion of pancuronium bromide (0.6 mgkg^−1^ h^−1^, i.v.). Rectal temperature was maintained at 37.0°C with a servo-controlled heating pad.

The microinjection pipettes were made from single-barrel glass pipettes with tip diameters of 25–30 µm (Catalog no. 626800; A-M Systems). They were filled with carbachol, a cholinergic agonist (carbamyl choline HCl, Sigma, St. Louis, MO) dissolved in 0.9% NaCl to 10 mM concentration with 2% of Pontamine sky blue dye (ICN Biomedicals, Aurora, OH) added to mark the injection sites. They were inserted into the dorsomedial pontine tegmentum aiming at the following stereotaxic coordinates: 3.1 mm caudal to bregma, 1.3 mm lateral from midline, and 8.2 mm below the cortical surface. The microinjections had a volume of 10 nl and were made over a period of 15–30 s by applying pressure to the fluid in the pipette while monitoring the movement of the meniscus with a calibrated microscope with 1 nl resolution. One pipette was used in each experiment and, once the site was verified to be effective at the beginning of the recording session, all subsequent injections were made at this one site. The pipettes for extracellular single cell recording were made from aluminosilicate glass (Catalog no. AF100-68-10, Sutter Instruments, Novato, CA) and had tips broken to a diameter of 2.5–3.0 µm. They were filled with 0.5 M Na acetate with 2% of Pontamine sky blue dye added to iontophoretically mark the recording sites. They were moved vertically using a digitally-controlled hydraulic microdrive (F. Haer and Co., Brunswick, ME).

The single cell recording pipette and the XII nerve, hippocampal and cortical EEG recording electrodes were connected to differential pre-amplifiers and amplifiers (NeuroLog modules 104 and 126, Digitimer Ltd., Hertfordshire, England). The signals were amplified and filtered at 100–3,000 Hz for single cell and XII nerve activity; 1–20 Hz for hippocampal activity; and 0.8–100 Hz for the cortical EEG. Arterial blood pressure was measured using a pressure transducer (P23 Db, Statham, Hato Rey, Puerto Rico). The raw and integrated XII nerve activity (time constant 100 ms; moving averager MA-821RSP, CWE Inc., Ardmore, PA), arterial blood pressure, inspiratory-expiratory CO_2_ difference (Micro Capnometer, Columbus Instruments, Columbus, OH) and event markers were digitized (Micro1401-3 data acquisition unit; Cambridge Electronic Design Ltd., Cambridge, England) and stored on a computer (Spike-2 software version 7; Cambridge Electronic Design Ltd.) using a sampling rate of 20,000 Hz for single cell recording, 5,000 Hz for the raw XII nerve activity, and 100 Hz for all other signals. The power of hippocampal activity in the theta frequency range (2.8–4 Hz in urethane-anesthetized rats [78]) was calculated offline in successive 10 s intervals (Spike-2 software).

### Experimental Protocol and Data Analysis

Our main goal was to explore catecholaminergic A1 and C1 cells of the VLM during REMS-like episodes. Most C1 cells are located in the lateral part of the lateral paragigantocellular region (LPGi), ventral to respiratory-modulated cells of the ventral respiratory group, and at antero-posterior levels between the rostral end of the LRN and the caudal margin of the facial nucleus (Mo7) [79], [80], [81], [82]. Further caudally, they are intermixed with noradrenergic A1 cells, which then predominate at more caudal levels where the LRN is the main cytoarchitectonically distinct structure. Accordingly, we searched for cells having spontaneous activity prior to carbachol injection at antero-posterior levels extending from −12.8 mm to −14.8 mm caudal relative to bregma according to a rat brain atlas [82], from 1.6 mm to 2.2 mm lateral from midline, and at depths below those at which we encountered respiratory-modulated neurons.

Once a stable recording from a single cell was established, we acquired a ∼1 min-long segment of minimally filtered record (1–3,000 Hz) for subsequent analysis of undistorted shape of action potentials and then, after at least 5 min of undisturbed baseline recording, we injected carbachol into the pons. Cell activity was monitored throughout the carbachol-induced REMS-like episode and for at least 5 min thereafter. The recording site was then marked with Pontamine blue deposit from the recording pipette (10 µA, 10 min, tip negative) and the pipette was withdrawn and placed in a different track. Up to 5 distinct recording sites were marked per experiment (mean: 2.5±1.2 (SD)), with a distance of at least 300 µm between the marked sites to allow for an unequivocal association of each site with the recorded cell.

At the end of the recording session, the animal received an additional dose of urethane (1.0 g/kg) and was intra-arterially perfused with 0.9% saline followed by 10% formalin. The brain was extracted, postfixed, cryoprotected in 30% sucrose, and the medulla and pons were cut into 25 µm coronal sections and those containing Pontamine blue deposit were collected. Pontine sections were sequentially mounted, stained with Neutral red, dehydrated, de-fatted and coverslipped, whereas medullary sections were subjected to immunohistochemistry for dopamine β-hydroxylase (DBH), a marker for catecholaminergic neurons. Immunohistochemical processing has been described previously [57]. In brief, free-floating sections were initially incubated in 1% borohydrate and then 70% ethyl alcohol mixed with 0.3% hydrogen peroxide to deactivate any residual formaldehyde and neutralize endogenous peroxidases. They were then incubated with DBH antibodies at 1∶500 concentration (catalog symbol: MAB308; Millipore, Billerica, MA) and the binding was visualized using biotinylated secondary antibodies tagged with horseradish peroxidase (Vectastatin Elite ABC reagent; Vector, Burlingame, CA). Horseradish peroxidase was reacted with 3 3' diaminobenzidine tetrahydrochloride resulting in a brown staining of DBH-positive (DBH^+^) neurons. The sections were then serially mounted and the one representing the center of each recording site was used to measure the distance between the center of the marked spot and the closest catecholaminergic cell bodies.

**Figure 1:**
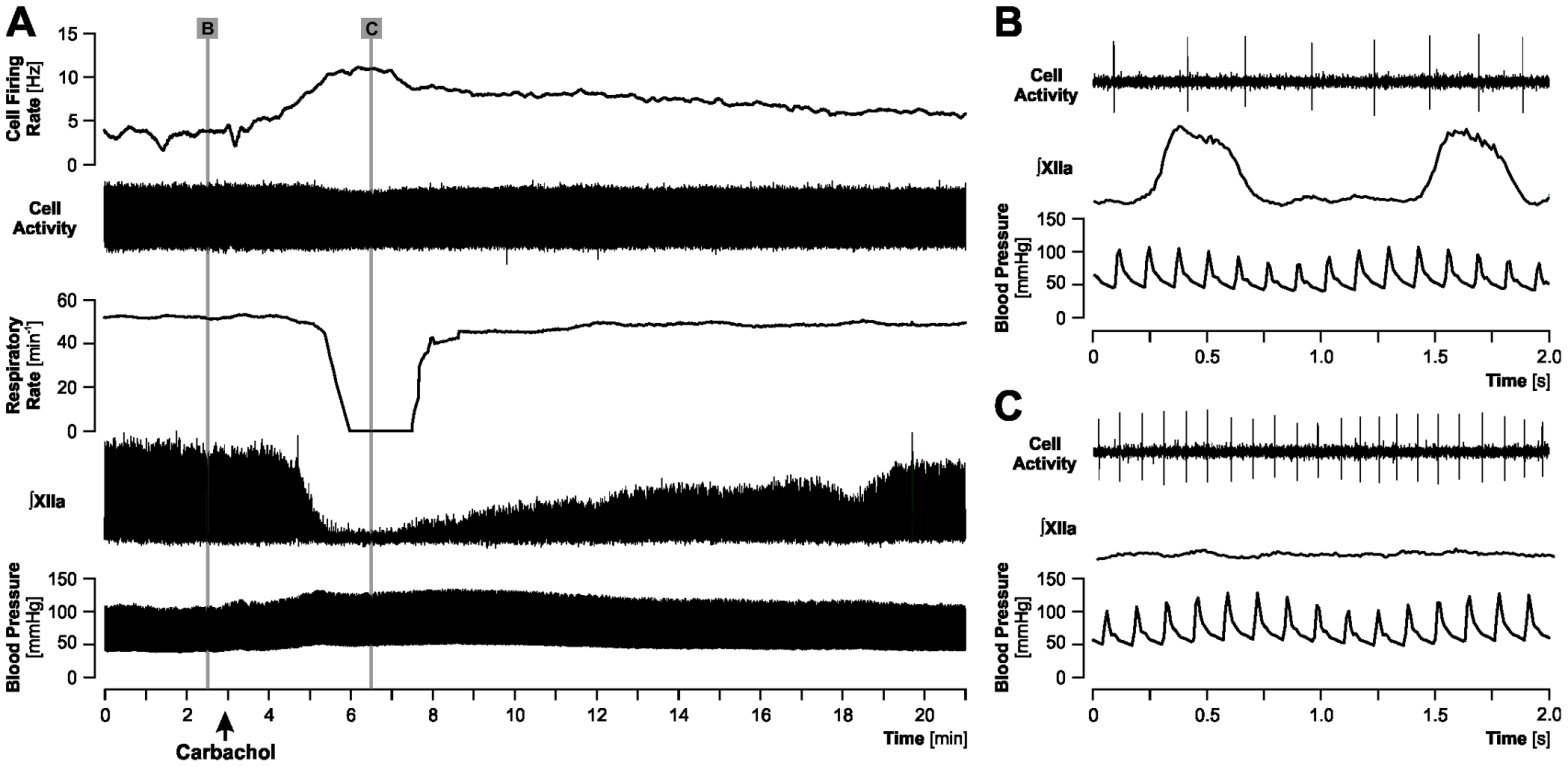
Example of a cell that was activated during a REMS-like episode. A: a 21 min-long record covering a period of baseline activity, carbachol-triggered REMS-like episode and recovery. The REMS-like episode is marked by the steep decline of integrated XII nerve activity (∫XIIa), ultimately leading to a transient disappearance of its inspiratory-modulated bursts, with a concurrent decline of the central respiratory rate and an increase of arterial blood pressure. The cell firing rate is more than doubled at the peak of the effect. Subsequently, both XII nerve activity and arterial blood pressure gradually recover, roughly in parallel to the decline of the firing rate of the cell. Carbachol was injected into the dorsomedial pontine tegmentum at the arrow (10 nl, 10 mM). B and C: expanded portions of the main record during the baseline period and at the peak of the effect, as indicated by the grey lines in A. The records show individual action potentials together with details of integrated XII nerve activity and blood pressure waveforms.

**Figure 2:**
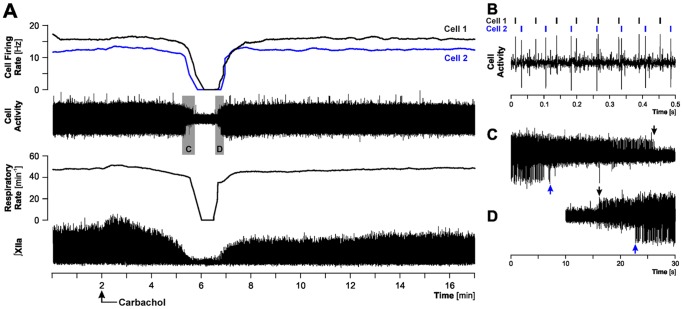
Example of a simultaneous recording from two cells that were silenced during a REMS-like episode. A: a 17 min-long record, with the period of REMS-like episode marked by a profound depression of XII nerve activity (∫XIIa) and a reduction of central respiratory rate that included a transient arrest of the central respiratory rhythm. During the episode, both cells are transiently silenced. Carbachol was injected into the dorsomedial pontine tegmentum at the arrow (10 nl, 10 mM). B: a segment of baseline activity at an expanded time scale showing different amplitudes and configurations of the action potentials generated by the two cells. C and D: expanded portions of the main record covering the periods when the two cells became silent and then resumed activity, as indicated by the grey areas superimposed on the cell activity trace in A. Arrows of the corresponding colors mark the offsets and onsets of firing for cells 1 and 2, as designated in A and B. The cell with a smaller action potential (cell 1) was silenced later than the larger cell (cell 2) at the beginning of the REMS-like episode and then resumed firing earlier than the cell with the larger action potential during the recovery.

Changes in cell firing rate associated with each REMS-like episode and other ancillary features of each cell’s activity were analyzed off-line in relation to the carbachol-induced changes in cortical and hippocampal activity and cardiorespiratory parameters. The changes in cortical EEG, hippocampal activity, XII nerve activity and respiratory rate that characterize REMS-like episodes triggered by pontine carbachol have been described in our earlier publications [65], [66], [67], [83], [84]. These changes were continuously monitored and used to determine whether carbachol injections were effective, which was a necessary prerequisite for subjecting the recorded single cell activity to off-line analysis. The amplitude of integrated XII nerve activity, instantaneous central respiratory rate, heart rate and mean arterial blood pressure were automatically derived from integrated XII nerve activity and arterial blood pressure records, respectively. For simplicity, our illustrations of cell activities during REMS-like episodes (Figs. 1 and 2) show only integrated XII nerve activity and respiratory rate or arterial blood pressure because their changes sufficiently delineate the onset of each episode and the subsequent recovery.

Action potentials were converted to standard pulses using threshold discrimination and peak detection features that are a part of the Spike-2 software. Mean cell firing rate was then calculated in successive 10 s intervals to determine the time course and pattern of firing rate changes over the entire duration of each REMS-like episode. The latencies to the onset of carbachol effect were measured between the onset of carbachol injection and the point when integrated XII nerve activity declined from the baseline by at least 10%. The durations of REMS-like episodes were measured between the point of the 10% decline and the point when XII nerve activity subsequently increased during the recovery by at least 10% of the total baseline amplitude. To characterize the effects of pontine carbachol on cell firing rate in a standardized way, the mean firing rate was calculated over a 1-min interval centered on the peak of the effect, during 5-min period prior to carbachol injection and another 5-min period after the recovery occurred. For the analysis of action potential durations, 5 successive action potentials recorded with broad-band filtering were averaged using the action potential peaks as triggers and then the durations of the negative deflection (main action potential) and positive deflection (afterpotential) were measured at the level of the half of the corresponding maximal amplitude relative to the isoelectric baseline ("half-widths"). The purpose of this analysis was to relate the action potential indices to the proximity of the recording site to DBH^+^ neurons in consideration of prior data showing that catecholaminergic cells have relatively longer action potentials and afterpotentials than most other cells recorded in the pontomedullary reticular formation [83], [85], [86], [87], [88], [89].

Cardiac modulation of cell activity was assessed by constructing cycle-triggered histograms of spike occurrence relative to the peak of the arterial blood pressure waveform. The histograms were constructed using 5 min of undisturbed recording of cell activity prior to carbachol injection. They had a bin width of 10 ms and a time base that covered approximately ±3 cardiac cycles relative to the triggering event. For cells whose histograms revealed the presence of a cardiac rhythm, the amplitude of modulation was measured as the difference in firing rate between the maximum and the minimum and its angular phase was determined between the peak of arterial blood pressure and the bin in the histogram that contained the lowest firing rate. With the average cardiac cycle length being about 130 ms, the histogram bin length of 10 ms allowed for the angular phase determination with a resolution of approximately ±14 degrees.

Each marked recording site was re-plotted onto the most appropriate standard cross-section of the medulla derived from a rat brain atlas [82]. The sites whose centers were less than 50 µm away from the nearest DBH^+^ cell (or a cluster of such cells) were operationally defined as those at which the recorded cell could be a catecholaminergic neuron and were designated as DBH^+^. All sites that did not fulfill this criterion were regarded as representing recordings from cells that were unlikely to be catecholaminergic and were operationally designated as DBH-negative (DBH^-^).

### Statistical Analysis

Statistical analysis was performed using SigmaPlot 12.0 software (Systat Software Inc., San Jose, CA). Normality of the distributions was tested using the Shapiro-Wilk test. Cell firing rates and other cardiorespiratory parameters at the baseline, the peak of carbachol effects and at the time of recovery were analyzed using repeated measures ANOVA with Holm-Sidak post-hoc comparisons. The results are presented as the mean ± standard deviation (SD). A P value less than 0.05 was considered significant. When data sets were not normally distributed, Mann-Whitney rank sum test was used for comparisons between two groups. Fisher-Exact test was used to compare proportions of cells expressing different features in different groups.

## Results

### Characteristics of Carbachol-induced REMS-like Episodes

While collecting data for the present study, we elicited 38 REMS-like episodes by injecting carbachol into the dorsomedial pons. The effective injection sites were localized within the dorsomedial pontine area that we described and illustrated in our earlier publications [65], [66], [67], [84]. Based on the time course of the decline of inspiratory modulation of XII nerve activity, the episodes occurred with a latency of 87±54 s after the onset of carbachol injection and lasted 380±220 s. In 25 of the 38 episodes, XII nerve activity was transiently abolished at the peak of the effect and in the remaining ones some respiratory-modulated activity was maintained throughout the episodes. The mean arterial blood pressure prior to carbachol injections was 62±12 mmHg, it increased during the episodes to 67±13 mmHg (P<0.000005, paired t-test re. baseline), and then returned to 61±12 mmHg after the recovery. The mean heart rate at baseline was 459±22 min^−1^, it decreased to 455±22 min^−1^ during the episodes (P<0.007, paired t-test re. baseline), and increased to 457±22 min^−1^ after the recovery.

### Cellular Behaviors During REMS-like Episodes: Cell Categories and Locations Relative to DBH Cells and LRN

We recorded from 50 VLM cells during one or more REMS-like episodes elicited by pontine carbachol. Twenty six recordings were from single cells and 12 from two adjacent cells that had sufficiently different amplitudes and configurations of their action potentials to allow for a reliable separation of their activities into distinct single-cell spike trains. Four single cell recordings and one two-cell recording were collected twice, with two pontine carbachol injections made successively at an interval of about 1 h. For all the 6 cells that were tested twice, the changes in cell activity during REMS-like episodes were qualitatively and quantitatively similar between the first and second test. The remaining 44 cells were recorded during one REMS-like episode each. To maintain a balanced design, the firing rates before, during and after the REMS-like episode collected from each of the 6 cells that were tested twice were averaged and then treated as one observation.

Our primary classification of cell behaviors during REMS-like episodes comprised four categories: (1) activated cells (n = 10); (2) silenced cells (n = 26); (3) suppressed, but not silenced, cells (n = 9); and (4) cells whose activity did not change in relation to the time course of the other characteristic effects despite otherwise clear evidence from observation of XII nerve activity, respiratory rate, cortical EEG and hippocampal activity that carbachol injections were effective (n = 5). Figure 1 shows an example of a cell that was activated. The cell firing rate begins to accelerate coincidentally with the onset of suppression of XII nerve activity and blood pressure increase. The peak firing rate coincides with the period of maximal suppression of XII nerve activity (transiently abolished) and then the firing rate gradually declines during the recovery of XII nerve activity and decline of arterial blood pressure. In contrast to this case, Fig. 2 shows an example of a simultaneous recording from two cells, both of which became silent during the REMS-like episode. The silencing occurred in temporal association with the suppression (transient abolition) of XII nerve activity. The cell with a smaller action potential stopped firing about 20 s later than the cell with the larger action potential (Fig. 2C) and then was the first one to resume firing during the recovery (Fig. 2D).

**Figure 3:**
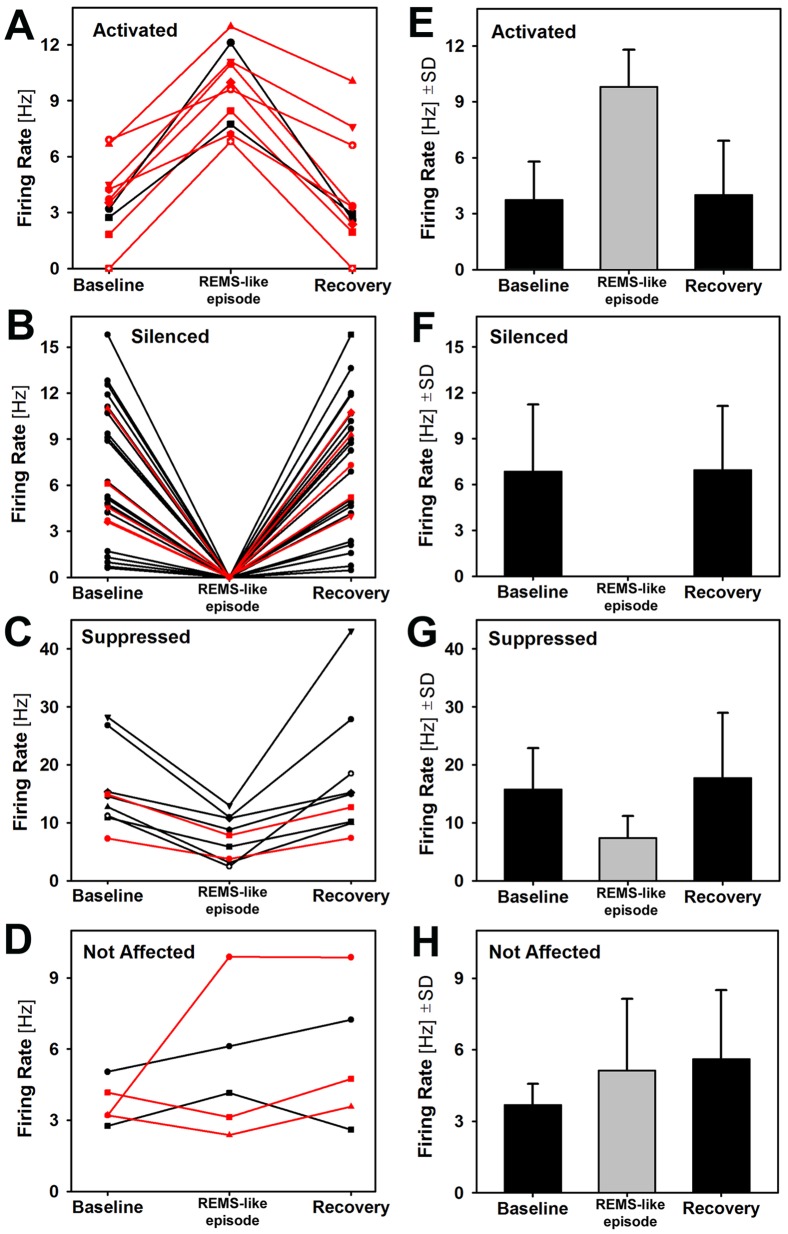
Individual and average firing rates within each cell category under the baseline conditions, during the REMS-like episodes, and following recovery. A–D: Firing rates of each of the 50 studied cells measured before, during and after REMS-like episode grouped by their different behaviors during the episode. The cells recorded at sites located closer than 50 µm from one or more DBH-positive (DBH^+^) neurons are marked by red lines. The lines representing firing rates of different cells end with different symbols to allow for tracking of each cell firing rate across the three conditions. The percentage of cells recorded near DBH^+^ neurons was significantly higher among the activated cells (80%) than among either the silenced (19%) or suppressed (22%) cells (P = 0.001 and P = 0.023 by Fisher-Exact test, with the odds ratios of 16 and 14, respectively). E-H: Mean firing rates before, during and after the REMS-like episodes for each of the four groups of cells. On the average, the firing rate changes between the baseline and maximal effect during the REMS-like episode were statistically significant for each of the affected groups, and the firing rate after the episode was not different from that during the baseline period. Activated cells had lower baseline firing rates than the silenced cells (P<0.038, unpaired t-test). The baseline firing rate of the latter was about twice lower than that of the suppressed cells (6.8±4.4 Hz vs. 15.8±7.1 Hz, P<0.0001), suggesting that the baseline firing rate determined whether a cell was silenced or only suppressed during the REMS-like episode.

Figure 3A–D shows the firing rates of each of the 50 studied cells and Fig. 3E–H the average firing rates before, during and after the REMS-like episode grouped by the nature of the cellular response. The three responsive cell groups had different baseline firing rates, the lowest for the activated cells, significantly higher for the silenced cells (6.8±4.4 Hz vs. 3.7±2.1 Hz; p = 0.038, unpaired t-test) and highest for the suppressed cells (15.9±7.1 Hz; p = 0.00009 vs. silenced cells). The average baseline firing rate of the 5 not affected cells was similar to that of the activated cells (3.7±0.9 Hz). Given that the silenced and suppressed groups were dominated by a relatively homogenous subset of cells that were recorded from the LRN (at least 26 out of 35 that were silenced or suppressed; see below), it is likely that the cells that were only suppressed during REMS-like episode were not entirely silenced as result of their baseline firing rates being higher than for the silenced cells.

**Figure 4:**
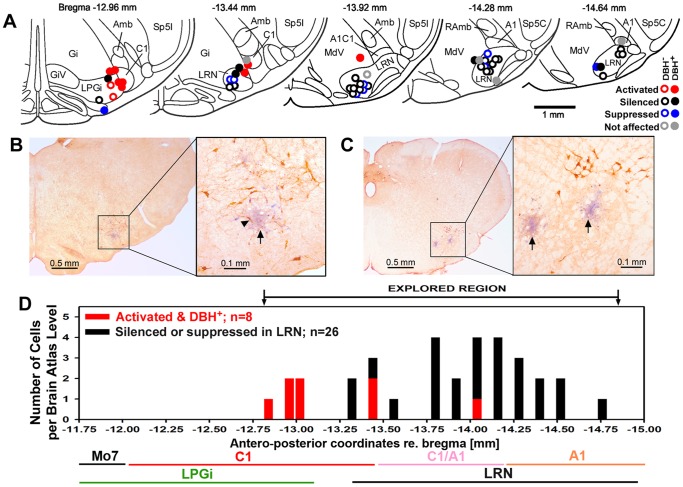
Cell behavior during the REMS-like episodes was related to the anatomical location of the recording site and its proximity to DBH-positive (DBH^+^) neurons. A: distribution of all recording sites superimposed onto a series of standard medullary sections from a rat brain atlas [82]. Different symbols indicate activated, silenced, suppressed and not affected cells and mark their relative proximity to one or more DBH^+^ neurons. Most activated cells (8 out of 10) were found adjacent to DBH^+^ cells in the C1 region, and the remaining 2 in the LPGi region. Most of the silenced or suppressed cells were located within the LRN (26 out of 35). The remaining 14 cells were a mixed group; some located near the edges of the LRN may have still belonged to the nucleus and some could be spontaneously active cells of the A1 group or other reticular formation neurons that became silenced or suppressed during REMS-like episodes. B and C: examples of marked recording sites (arrows) in sections immunohistochemically labeled for DBH. The recording site in B was localized in the rostral part of the explored VLM region and within less than 50 µm from several DBH^+^ neurons (arrowhead points to a DBH-labeled cell located closest to the center of the recording site marked by Pontamine blue deposit). The two recording sites in C were localized more caudally, within or near the LRN, and clearly away from more dorsally located DBH^+^ neurons (noradrenergic A1 group). D: distribution of the activated, silenced and suppressed cells, as assigned to one of the 17 different antero-posterior levels represented in the rat brain atlas that we used as reference [82]. The lines under the abscissa mark the levels corresponding to the C1, A1/C1 and LPGi regions and the LRN, as marked in the same atlas. The diagram shows the antero-posterio range of the VLM that we explored and its rostral extension up to the facial motor nucleus (Mo7) that contains the rostral part of the adrenergic C1 group but was not explored in our study. Abbreviations: Amb - nucleus ambiguous; Gi – gigantocellular reticular region; GiV – gigantocellular ventral reticular region; MdV – ventral medullary reticular region; RAmb - retroambiguus nucleus; Sp5C – spinal trigeminal nucleus, caudal division; Sp5I - spinal trigeminal nucleus, interpolar division.

The cell groups initially classified on the basis of their behavior during the REMS-like episodes were differentially distributed within the explored region of the VLM and in relation to the presence of DBH^+^ cells near the recording site. Figure 4A shows the anatomical distribution of all 50 recording sites superimposed onto selected standard cross-sections through the lower medulla derived from a rat brain atlas [82]. Most of the activated cells, 9 out of 10, were recorded at relatively rostral levels of the explored region. Furthermore, 8 out of the 10 were located at sites adjacent to DBH^+^ neurons (Fig. 4B). Indeed, all these 8 cells were recorded at sites located within the area designated in our reference brain atlas as the C1 or A1/C1 region and no such a cell was found at more caudal levels where noradrenergic A1 cells predominate (Fig. 4C and D). The remaining two activated cells were recorded at the rostralmost part of the explored VLM and relatively medially. These two recording sites were localized in the LPGi region (Fig. 4A), as designated in our reference brain atlas [82].

The silenced and suppressed cells collectively represented the largest group in our study, with the suppressed cells interspersed among the silenced ones. Of particular note about this group is that 26 of the 35 cells of this combined category were localized clearly within the LRN and at a considerable distance from any DBH^+^ neurons. Thus, there was high level of certainty that they belonged to the precerebellar LRN. Most of the remaining 9 cells were recorded at sites surrounding the LRN. Seven were located near one or more DBH^+^ neurons (5 of those were silenced), one at a distance from such cells and near the border between the LPGi region and the inferior olive, and another one also away from DBH^+^ neurons and near the ventral border of the LRN. Thus, these 9 cells were probably a mixed group; some could still belong to the LRN but we have not classified them as such because of their peripheral location relative to the nucleus. Some could be noradrenergic cells of the A1 group. However, considering that many A1 neurons occur in clusters located dorsal to the LRN (Fig. 4C), we were surprised to have found only 3 spontaneously active cells located at the levels appropriate for the A1 group that were recorded near DBH^+^ neurons (2 were silenced and 1 was suppressed). Indeed, we believe that our population of cells that we studied was relatively "enriched" with LRN neurons because our other target, the noradrenergic A1 neurons, must have been silent under our baseline conditions. Should they be active, many electrode tracks would end with a recording from one of these cells and then marking of the site, which would result in relatively fewer neurons recorded in the LRN.

The anatomical distribution of the activated cells recorded at DBH^+^ sites and the silenced or suppressed cells recorded within the LRN is shown in Fig. 4D in relation to the levels corresponding to the LRN, A1, A1/C1 and C1 groups. The antero-posterior location of each recording site is assigned the closest level selected from the 17 standard plates spaced by approximately 120 µm that cover the VLM region that we explored and are included in our reference brain atlas [82]. All these silenced and suppressed neurons were found at the levels corresponding to the LRN. In contrast, the close proximity to DBH^+^ neurons of most activated cells and the location of such sites at the rostral end of the explored region support the conclusion that the activated cells were adrenergic neurons of the C1 group. Our findings also suggest that noradrenergic A1 cells were silenced, but the population of putative A1 cells tested was too small to draw a firm conclusion.

It is of note that in none of the 12 instances when we recorded simultaneously from two cells during the REMS-like episodes did we observed a case when the behavior of one cell would be opposite to that of the other, such as one cell being activated and the other suppressed or silenced. Nor have we observed at any recording site cases when a cell silent under the baseline condition would be recruited following carbachol injections while the originally investigated cell became suppressed or silenced. In 10 of the 12 cases with two cells recorded simultaneously, both were suppressed or silenced (as in Fig. 2), in one case the cell originally selected for the test was activated and then another initially silent one became active during the episode, and in one case one cell was suppressed and the other was not affected. The absence of evidence for adjacent location of cells having opposite changes in firing rate during REMS-like episodes suggests that, in the part of the VLM that we explored, mutually inhibitory interactions among local cells were not common. Rather, it appears that the suppressed/silenced and activated cells aggregated in clusters, with the changes of activity during the episodes being imparted on them through afferent projections from regions located elsewhere.

### Relationship of Cell Behavior Characteristics and Location to Action Potential and Afterpotential Durations

**Figure 5:**
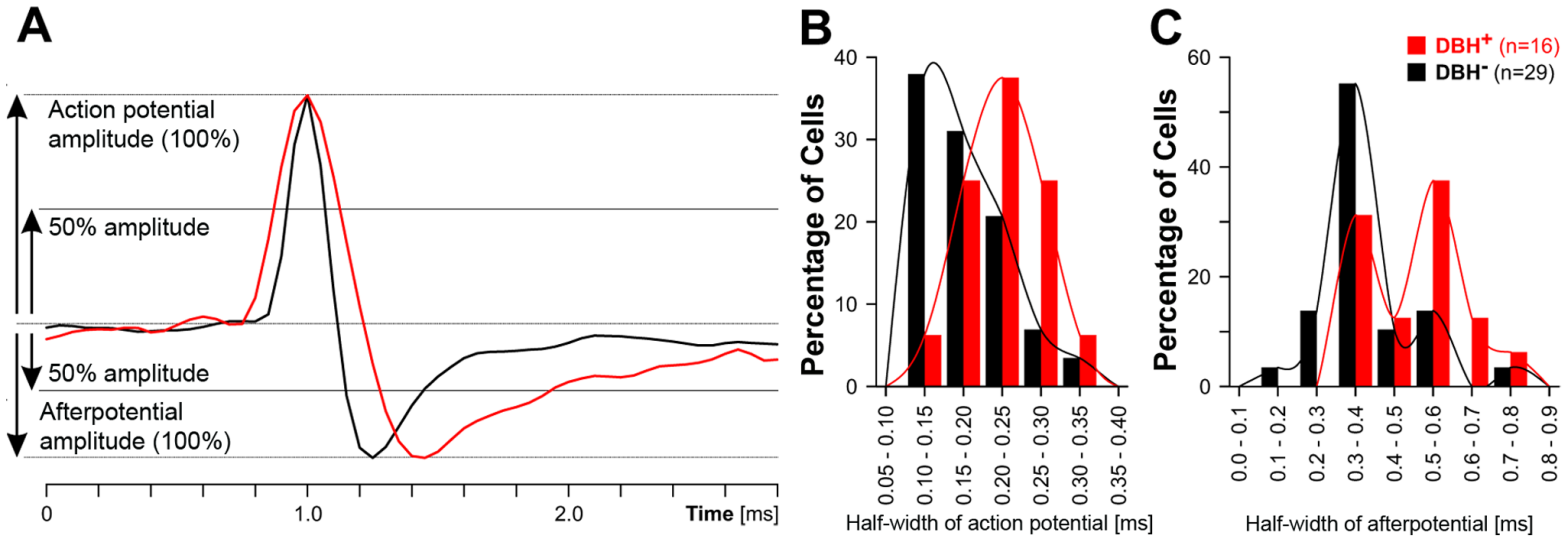
Cells recorded near DBH-positive (DBH^+^) neurons had significantly longer action potentials and afterpotentials than those recorded at a distance from DBH^+^ cells. A: the scheme explaining how the half-widths of action potentials and afterpotentials were measured. Two spike waveforms are superimposed, one typical of a cell with a fast action potential and another for a cell with a slow action potential. The histograms in B and C show that both the action potentials and afterpotentials had longer half-widths for the cells recorded at sites containing DBH^+^ neurons than for the cells located at a distance from such sites. Since the majority of cells that were activated were found in the rostral part of the explored region of the VLM and adjacent to DBH^+^ neurons, the spike duration data support the conclusion that most of the cells activated during REMS-like episodes were the adrenergic cells of the C1 group.

Aminergic and cholinergic brainstem cells have relatively longer-lasting action potentials and afterpotentials than most other cells in the pontomedullary reticular formation [83], [85], [86], [87], [88], [89]. Analysis of the half-widths of the spike waveforms of all recorded cells was undertaken to further test whether the activated cells and/or the cells recorded at sites where we found DBH^+^ neurons were likely to be aminergic. The measurements were taken using the approach explained in Fig. 5A and were derived from minimally filtered records of cell activity (see Methods) to minimize the distortion of the waveforms. Out of the 50 cells recorded during the REMS-like episodes, 5 could not be subjected to this analysis; 2 because unfiltered records were not available and 3 because their action potentials were too complex.

In a comparison between the cells recorded near DBH^+^ neurons and those recorded at a distance from any such cells, the former had both significantly longer actions potentials and afterpotentials (Table 1). Indeed, despite the limitations of inferring about the neurochemical phenotype of the studied cell from the phenotype of cells present near the recording site, both indices had bimodal distributions, with two distinct peaks made up mainly of the cells recorded at DBH^+^ and DBH^-^ sites, respectively (Fig. 5B and C). Similarly, when activated cells were compared to the silenced/suppressed cells irrespectively of the proximity of the recording site to DBH^+^ neurons, activated cells had significantly longer action potentials and afterpotentials than the silenced and suppressed cells combined (Table 1). This reflected the high preponderance of cells recorded at DBH^+^ sites among the activated cells (8 out of 9 subjected to spike shape analysis) and the relative scarcity of cells recorded at DBH^+^ sites among those that were silenced, suppressed or not affected (6 out of the 33 analyzed). Thus, both ways of grouping the cells yielded results supporting the conclusion that most of the activated cells and most of those recorded at sites containing DBH^+^ neurons were likely to be aminergic.

**Table 1:** Relationship between action potential and afterpotential half-width durations and the proximity of the recording site to DBH^+^ neurons and cell behavior during the REMS-like episodes.

Cell category	Near DBH^+^neurons	No DBH^+^ neurons nearby	P level*	Activated cells	Silenced orsuppressed cells	P level*
Number of cells	16	29		8	32	
Action potential (mean ±SD; ms)	0.22±0.04	0.18±0.05	0.008	0.23±0.03	0.19±0.05	0.019
Afterpotential (mean ±SD; ms)	0.49±0.10	0.38±0.12	0.001	0.49±0.10	0.40±0.13	0.029

* -All significance levels determined by Mann-Whitney rank sum test.

### Cardiac Modulation of Cell Activity

Most adrenergic C1 neurons are inhibited when arterial blood pressure is increased and, as a reflection of this inhibition, their spontaneous activity exhibits cardiac modulation [79], [90], [91], [92], [93], [94]. Therefore, to further assess whether our population of cells activated during REMS-like episodes had this property expected of C1 neurons, we examined cardiac modulation of the baseline firing rate of 48 out of the 50 cells that we recorded in this study (for 2 cells, the baseline firing rate was too low to allow for conclusive analysis).

**Figure 6:**
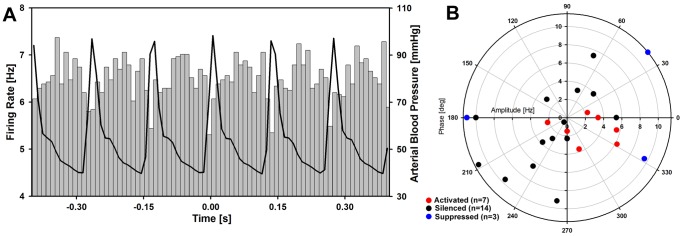
Cardiac modulation was detected in most cells activated during REMS-like episodes and in about a half of those that were silenced or suppressed. A: example of a histogram of cell firing rate triggered from the peak of arterial blood pressure waveform. The concurrently averaged blood pressure trace is superimposed over the histogram of cell firing rate. The cell was activated during the REMS-like episode and was recorded at a site containing DBH^+^ neurons (amplitude of cardiac modulation: 1.6 Hz, angular phase of the lowest firing rate relative to the peak of arterial blood pressure: +14 deg). B: polar diagram illustrating the distribution of amplitudes and phases of cardiac modulation of all cells in which such modulation was detected (7 out of 9 activated cells, 14 out of 24 silenced cells, and 3 out of 9 suppressed cells). Notably, for all 5 cells that were activated during the REMS-like episodes and recorded at sites containing DBH^+^ neurons, the angular phase of the minimum of their firing rate was close to the peak of arterial blood pressure (red symbols in and near the lower right quadrant), whereas the silenced and suppressed cells recorded from the LRN and the two activated cells that were recorded in the LPGi region had widely scattered phase angles. This is consistent with the first group being inhibited by stimulation of arterial baroreceptors and the other cells being affected by pulse pressure through more complex pathways.

Owing to the peak arterial blood pressure being maintained around 100 mmHg, none of the studied cells had an overt cardiac modulation that would be noticeable by direct observation (cf. [79], [94]). However, cycle-triggered averaging revealed that 7 out of 9 activated cells, 14 out of 24 silenced cells, 3 out of 9 suppressed cells, and none out of 5 not affected cells had cardiac modulation. Thus, the presence of cardiac modulation was not a unique feature associated with any one type of behavior during REMS-like episodes, but there was a trend towards a higher proportion of cells with cardiac modulation among the activated cells. Furthermore, 5 out of the 7 cells that were activated during REMS-like episodes and were recorded at DBH^+^ sites had the angular phase of the minimum of their firing rate closely aligned with the peak of arterial blood pressure, whereas all the remaining cells in which cardiac modulation was detected had widely scattered phase angles (this group includes the two activated cells that were recorded in the LPGi region, 14 silenced and 3 suppressed cells). Figure 6A shows an example of a cell that had reduced firing rate in association with the rising slope of arterial blood pressure, and Fig. 6B shows the polar plot with the amplitudes and phases of cardiac modulation for all cells in which such a modulation was detected. The cell in Fig. 6A was activated during the REMS-like episode and was recorded at a site containing DBH^+^ neurons. Thus, the analysis of cardiac modulation yielded results consistent with an inhibitory effect of arterial baroreceptors on cells that were activated during the REMS-like episodes. For all other cell categories, the incidence of cardiac modulation tended to be lower and the angular phase of the modulation, when present, suggested that the input related to pulse pressure reached them through more complex pathways.

## Discussion

We characterized the behavior of two major cell groups located in the caudal and intermediate VLM during pharmacologically induced REM sleep-like episodes, the adrenergic cells of the C1 group and the precerebellar cells of the LRN. We determined that C1 cells are activated, whereas LRN cells are silenced or suppressed during the episodes. To our knowledge, our study provides the first ever direct insight into the behavior of C1 and LRN neurons during REMS.

Adrenergic C1 neurons play an important role in driving sympathetic output and, through their ascending projections may control the generation of REMS. Our finding that they are activated during REMS-like state provides a mechanistic explanation for arterial blood pressure increases that occur during transitions from non-REMS to REMS. The LRN neurons are a part of an important spino-reticulo-cerebellar pathway in which the intended and actual movement trajectories are compared and corrective signals are generated to ensure a smooth and precise execution of motor tasks. We suggest that the REMS-related suppression of activity in LRN neurons may be of particular relevance in the condition known as the REMS behavior disorder (RBD) in which patients can execute complex motor task while they are in REMS based on all other electrophysiologic criteria.

Before we discuss our findings and their implications, we briefly discuss the features of the model of REMS that we used when compared to natural REMS.

### Advantages and Limitations of the Anesthetized Rat Carbachol Model of REMS

The REMS-like state elicited by pontine carbachol injections in anesthetized, paralyzed and artificially ventilated rats does not fully replicate all features of natural REMS but the two bear many important similarities. The location of the effective sites for eliciting the REMS-like state by pontine carbachol (or bicuculline) in urethane-anesthetized rats [65], [67], [95] corresponds well to the location of effective sites in chronically instrumented rats [68], [69], [70], [71] (injections volumes in behaving rats are by necessity relatively larger, which limits their spatial resolution). Activation of cells in the wake-promoting region of the posterior, lateral hypothalamus abolishes the ability of pontine carbachol to elicit REMS-like state in urethane-anesthetized rats [66] and optogenetic or pharmacological activation of cells in the same hypothalamic region suppresses generation of sleep, including REMS, in behaving rats [72], [73]. Carbachol-induced REMS-like state in anesthetized rats is characterized by activation of cortical EEG and the appearance of theta-like rhythm in the hippocampus [66], [78], as is also typical of natural REMS. The frequency of the theta-like rhythm elicited in urethane-anesthetized rats (2.8–4.0 Hz) is slower than the REMS-related or sensory-evoked theta rhythm in unanesthetized, behaving rats (6–9 Hz) but both rhythms are generated within the same ponto-septo-hippocampal network and similarly respond to pharmacological manipulations and sensory stimulation [96], [97].

Noradrenergic cells of the LC and pontine A5 group are silenced during carbachol-induced REMS-like state in anesthetized rats [65], [83] and the same is the case during natural REMS [13], [19], [98]. Ventral medullary inspiratory cells have unchanged or increased activity during carbachol-induced REMS-like state in anesthetized rats and decerebrate cats [99], [100] and these cells are also activated during natural REMS [101], [102]. Caudal medullary serotonergic cells are silenced during carbachol-induced REMS-like state in decerebrate cats [39] and during natural REMS in behaving cats [36], [37]. Furthermore, the neurochemical mechanisms of REMS-related suppression of activity in orofacial motoneurons are similar when investigated in carbachol models of REMS [84], [103], [104] and during natural REMS [105], [106], [107]; see [108] for an overview).

Thus, the list of similarities at both the systemic and cellular levels between natural REMS and carbachol-induced REMS-like state is long and supportive of the contention that the pontine carbachol injections activate a major part of the brainstem network responsible for the generation of natural REMS, but the two states are not fully equivalent. What the carbachol models of REMS are consistently lacking is the phasic components of REMS, such as phasic bursts of motoneuronal activation (muscle twitches), transient accelerations of respiratory rate and transient surges of arterial blood pressure. We have previously argued that the absence of phasic phenomena may be related to the mode of state initiation and maintenance. In the carbachol models, a cholinergic agonist is deposited into the REMS triggering zone in the pons and exerts its action steadily over a certain period of time whereas natural REMS occurs as a result of natural activation of a network of neurons responsible for the initiation and maintenance of this state which may rapidly wax and wane [74]. Generation of phasic events is probably additionally suppressed by anesthesia, as is the regulation of the duration of the episodes. In decerebrate, unanesthetized rats, phasic muscle twitches do not occur during the carbachol-induced REMS-like episodes of atonia but the episodes last longer than in urethane-anesthetized rats and often have abrupt terminations [109]. As with natural REMS, the duration of the episodes is variable under both anesthetized and decerebrate conditions and does not obviously depend on the amount of carbachol injected provided that the drug does not significantly spread into different reticular regions that exert opposite effects on the REMS-related pontine network [109], [110]; discussed in [65]. Thus, the carbachol-induced REMS-like state elicited under urethane anesthesia cannot be used to study the mechanisms of phasic events of REMS, but by all measures obtained to date well replicates the tonic aspects of network activation that leads to the generation and maintenance of REMS episodes.

Additionally, it is important to note that our study was conducted under neuromuscular paralysis and with artificial ventilation at a constant rate and volume. Consequently, any cellular and cardiorespiratory changes observed during the REMS-like state can be interpreted as resulting from activation of the central network responsible for the generation of REMS, rather than being caused by secondary effects due to muscle relaxation or changes in ventilation that occur during REMS in behaving animals. We see this as an advantage of our model when used to study cells that are a part of the REMS network that controls the cardiorespiratory and motor systems.

### Adrenergic C1 Cells are Activated During the REMS-like State

Most of the cells that were activated during the REMS-like state were found in the rostral part of the VLM that we explored. This region contains adrenergic cells of the C1 group, and most of the sites at which we found activated cells contained DBH^+^ neurons. The activated cells had longer action potentials and afterpotentials than most other cells, as is typical of aminergic neurons [83], [85], [86], [87], [88]. In addition, they had detectable cardiac rhythmicity with a phase relationship to arterial blood pressure suggestive of an inhibitory effect of pulse pressure on cell activity, as is typical of C1 neurons with axonal projections to the spinal cord or hypothalamus [79], [90], [91], [92], [93], [94]. Collectively, this combination of features leads us to conclude that the activated cells most likely represented adrenergic cells of the C1 group.

To date, most cells tonically activated during REMS have been recorded from relatively more rostral and more medial regions of the medullary reticular formation in cats [31], [32], [33], [34], [35]. A recent study in chronically instrumented, REMS-deprived and head-restrained rats found that the population of REMS-on medullary reticular neurons extends further caudal and lateral than in earlier reports [40]. Although the actual recording sites were not shown, these cells appear to have been recorded from the LPGi region just caudal to the Mo7, thus at slightly more rostral levels than those that we explored in our study (cf. Fig. 4D). However, both levels contain adrenergic C1 neurons [80], [94]. Within this region, spinally projecting C1 neurons predominate immediately caudal to the Mo7, whereas at the levels from 600 µm caudal to the Mo7 to the rostral end of the LRN spinally projecting C1 neurons decrease in numbers and are replaced by C1 neurons that have axonal projections to the posterior lateral hypothalamus [80], [94]. Since we explored only the caudal half of the C1 region (cf. Fig. 4A and D), our population of REMS-like state-activated cells could include both those with ascending and those with descending projections. It is of note that, despite different axonal projections, both groups are inhibited by stimulation of arterial baroreceptors [94]. The region also contains a third sub-population of neurons that are likely to be adrenergic and have axonal projections to the LC [44], [93], [111]. However, in contrast to the C1 cells with either spinal or hypothalamic projections, these cells do not have cardiac modulation and are only weakly inhibited by experimentally increased arterial blood pressure [93]. In our study, among the 8 REMS-like state-activated cells that were recorded at DBH^+^ sites, two had no cardiac modulation. Thus, it is possible that our group of activated cells included LC-projecting C1 neurons. Collectively, our findings identify the adrenergic C1 cells as a major population of neurons in the region between the Mo7 and LRN that has a REMS-on pattern of activity.

Based on our data, many REMS-on cells recorded by Sirieix et al. [40] could be adrenergic C1 neurons, and predominantly those with spinal projections to preganglionic sympathetic neurons. However, these authors proposed that their REMS-on neurons represented a population of inhibitory (GABA-ergic or glycinergic) interneurons whose function would be to inhibit motoneurons or noradrenergic LC neurons during REMS. This interpretation was based on juxtaposition of cell recording data with results from separate tract-tracing and immunohistochemical experiments in which the LPGi region located caudal to the Mo7 was found to contain GABA-ergic cells and cells that had axonal projections to either the Mo7 or LC. Furthermore, in the same study, the authors suggested that adrenergic C1 neurons may belong to the REMS-off category on the basis of extrapolation from electrophysiological studies of pontine noradrenergic neurons that have well established REMS-off patterns [13], [83], [98]. However, the exact location of the recording sites relative to the location of cells with any of these distinct phenotypes was not determined and an attempt to classify the cells based on the shapes and durations of their action potentials did not yield conclusive results. Furthermore, none of the two prior attempts to use c-Fos expression to indirectly assess the impact of REMS of C1 cell activity was supportive of C1 cells being suppressed during REMS. One of these studies indicated that the C1 region contains a population of cells that have axonal projections to the LC and are activated following a prolonged period of REMS [112]. The other study found insignificant decline of c-Fos expression in rostral VLM neurons containing tyrosine hydroxylase following prolonged periods of REMS-like state elicited by pontine carbachol which was in contrast to significant c-Fos declines detected in noradrenergic neurons of the pontine A5, A7 and sub-coeruleus groups [110]. Accordingly, in consideration of these earlier results and those from our present study, we propose that C1 neurons are a major group of REMS-on neurons in the part of the LPGi region that contains adrenergic cells.

Our finding that adrenergic C1 neurons are activated during REMS-like state provides a mechanistic explanation for the mean arterial blood pressure increases that occur during transitions from slow-wave sleep to REMS [113], [114], [115]. Indeed, the average blood pressure increases that we observed during REMS-like episodes were significant and of a similar magnitude as those observed on the average during REMS [113], [114], [115] (as discussed in the preceding section, the model that we used does not generate phasic events of REMS; accordingly, we cannot comment on blood pressure variability that is another characteristic feature of natural REMS but is absent from the carbachol models). Blood pressure increases occurring concurrently with activation of adrenergic C1 neurons are consistent with our interpretation that the population of adrenergic C1 neurons that are activated during REMS-like state includes the spinally projecting presympathetic neurons in addition to those with ascending projections to the hypothalamus and LC. This interpretation is consistent with two recent studies showing that selective optogenetic stimulation of C1 neurons elicits glutamatergic activation of LC neurons and increases arterial blood pressure [47], [111]. Since activation of LC neurons suppresses generation of REMS [116], [117], we suggest that VLM adrenergic REMS-on cells may contribute to the termination of REMS episodes, rather than to their maintenance.

### LRN Cells are Silenced or Suppressed During REMS-like State

Our interpretation that a substantial proportion of cells that were silenced or suppressed were the cells of the precerebellar LRN was conservative in that we included in this category only those suppressed and silenced cells that were recorded clearly within the nucleus and away from any DBH^+^ neurons. Still such cells represented a majority, 26 out of 35 silenced or suppressed neurons, with the remaining 9 recorded near the edges of the LRN and of those 7 at DBH^+^ sites. These proportions convincingly indicate that LRN cells are suppressed or silenced during the REMS-like episodes. They also suggest that some noradrenergic A1 neurons are suppressed or silenced, although we regard this conclusion as a tentative one because 3 out of the 5 not affected cells were also recorded at levels appropriate for A1 neurons and at sites adjacent to DBH^+^ neurons. In addition, our earlier c-Fos study did not support the conclusion that noradrenergic A1 neurons are suppressed during REMS-like state elicited by pontine carbachol [110]. Thus, we currently think that many noradrenergic A1 neurons are silent in our model, which would explain the relative scarcity of putative cells of this type in our sample. Accordingly, the verdict is open as to whether A1 neurons are suppressed or silenced, as is typical of pontine noradrenergic neurons.

In contrast to the uncertainty about A1 neurons, our evidence for suppression of activity in LRN neuron during REMS-like state is supported by recording from many cells. To our knowledge, this is the first direct electrophysiological demonstration that LRN neurons are consistently affected by REMS and that the effect is a suppression of their activity. To date, REMS-related suppression of transmission has been reported for trigeminal sensory pathways and it was explained, at least in part, by a state-dependent primary afferent depolarization [118], [119]. In contrast, activity of dorsal spinocerebellar and spinoreticular tract neurons was reported to be suppressed, increased or unchanged [120], [121], [122], [123], [124], and the tactile receptive fields and tactile responsiveness of a majority of spinal dorsal horn neurons were increased during REMS [125]. The variability of these results may be caused by a convergence on these neurons of possibly opposite effects mediated by central REMS-related pathways and changes in peripheral inputs associated with the atonia of REMS. Since our study was conducted in paralyzed and artificially ventilated animals, the suppression of LRN cell activity that we found can be ascribed with a high degree of confidence to the central effects of REMS-like state on the excitability of LRN neurons.

Suppression of LRN cell activity during REMS-like state may be caused by an active, state-dependent inhibition or a state-dependent withdrawal of excitatory effects (disfacilitation). Additional studies will be needed to distinguish between these two possibilities. Regardless of the underlying mechanism, our findings provide evidence of suppression of transmission in an important spino-reticulo-cerebellar pathway that is functionally and anatomically different from the dorsal pathways that have been studied to date. Specifically, the LRN receives information from flexion-reflex afferents that is transmitted to the nucleus through the ventral funiculi of the spinal cord and integrates it with inputs from the vestibular system and those related to the descending motor commands [58], [59], [64], [126], [127]. This combined information is sent via axons of LRN neurons (mossy fibers) to the cerebellar cortex, cerebellar nuclei and pontomedullary reticular formation and is used to achieve a smooth and precise execution of movements [128]. Under most physiologic conditions, the REMS-related suppression of LRN neuronal activity may be relatively unimportant due to the general suppression of motor activity during REMS. However, it has been hypothesized that altered transmission in the pathways that carry information about the gravitational forces may impact brain activity during REMS, including the perception and interpretation of body position [129]. Thus, it is possible that suppressed transmission through the LRN contributes to the often “bizarre” perception of movements during dreams and facilitates the occurrence of phasic body movements during natural REMS that are occasionally very large. The REMS-related suppression of transmission through the LRN also may play a significant role in RBD. In RBD patients, suppression of motor activity during REMS is impaired due to degenerative processes within the REMS-generating network and the patients move in a manner suggesting that they act-out their dreams [130], [131], [132]. However, the movements are imprecise, often exaggerated, suggesting that they lack the proper feedback control through the cerebellar circuits. Thus, our finding that LRN neurons are suppressed during REMS may be of particular relevance for the pathophysiology of RBD.

### Conclusions

We determined the behavior of two major cell groups located in the intermediate and caudal part of the VLM, the adrenergic cells of the C1 group and the precerebellar cells of the LRN, during pharmacologically induced REMS-like episodes. The C1 neurons play a major role in the regulation of sympathetic output and may also be an important part of the central network responsible for the generation of REMS. Our data suggest that activation of these cells is, on the one hand, responsible for sympathetic activation during REMS and, on the other hand, contributes to the termination of REMS episodes. Suppression of LRN neuronal activity during the REMS may alter the spatial representation of body position. This, in turn, may be an important cause of the often unusual dream imagery of movements during REMS and may contribute to the generation of exaggerated movements in RBD.
